# The cost of doubt: assessing the association between attributional ambiguity and mental health

**DOI:** 10.1186/s12889-024-17664-1

**Published:** 2024-01-09

**Authors:** Adolfo G. Cuevas, David R. Williams, Danielle M. Krobath, Adiammi Lyngdoh, Fatoumata Kaba-Diakité, Jennifer D. Allen

**Affiliations:** 1 Department of Social and Behavioral Sciences, New York University School of Global Public Health, 708 Broadway, New York, NY 10003 USA; 2 Center for Anti-Racism, Social Justice, and Public Health, New York University School of Global Public Health, New York, NY USA; 3 Department of Social and Behavioral Sciences, Harvard T.H. Chan School of Public Health, Boston, MA USA; 4 Department of African and African American Studies, Harvard University, Cambridge, MA England; 5 Friedman School of Nutrition Science and Policy, Tufts University, Boston, MA USA; 6 Eliot-Pearson Department of Child Study and Human Development, Tufts University, Medford, MA USA; 7 Department of Community Health, Tufts University, Medford, MA USA

**Keywords:** Attributional ambiguity, Discrimination, Mental health

## Abstract

**Objective:**

To quantify the association between attributional ambiguity–the uncertainty of whether an experience is discrimination–and mental health.

**Methods:**

Using a nationally representative sample of U.S. adults recruited through an online survey by Ipsos (April 23 and May 3, 2021), attributional ambiguity was quantified by asking participants if they experienced anything in the past 6 months that they were unsure was discrimination. The survey also assessed the degree to which these experiences caused participants to feel bothered and to ruminate on them. Multiple linear regression models were used to analyze associations between attributional ambiguity and depressive symptoms and mental health status.

**Results:**

Black and Hispanic participants reported higher rates of attributional ambiguity than White participants. Experiencing attributional ambiguity was associated with higher levels of depressive symptoms and poorer self-reported mental health status. Among those who reported attributional ambiguity, increases in bother and rumination scores were positively associated with depressive symptoms.

**Conclusion:**

Attributional ambiguity is an important yet overlooked social determinant of mental health. More research is needed to fully understand the impact of this stressor on population health, particularly among minoritized populations.

## Introduction

Discrimination is a well-recognized social determinant of mental health that influences psychological distress, depressive symptoms, and anxiety [1–4]. However, studies have focused primarily on self-reported perceived discrimination. However, this body of work largely assumes that the discrimination experience involves clear and straightforward experiences of unfair treatment [1, 5, 6]. Attributional ambiguity, also known as ambiguous discrimination), refers to negative experiences that an individual cannot directly pinpoint as discrimination, thus leaving room for interpretation of its causes [5]. It can also include experiences that are very clearly attributable to bias, but the target is uncertain about the specific reason for the bias. For example, The subtlety of racial bias can be so pronounced that it leads individuals to second-guess the intent behind these experiences [7]. In such situations, it becomes challenging for the individual to address the issue or find a resolution.

Attributional ambiguity can impact health, Experimental investigations into attributional ambiguity’s influence on health have rigorously probed how situations fraught with ambiguity and vague causal attributions can exert a profound impact on both the psychological and physical well-being of the targeted individuals. These studies routinely involve the purposeful manipulation of attributional ambiguity across diverse health-related contexts and a meticulous assessment of its effects on mental health and related outcomes. For instance, Crocker and Major [8] illustrated that Black participants who were made visible to an evaluator were more prone to attribute both negative and positive evaluations to prejudice. Notably, being made visible by the evaluator acted as a protective shield for the self-esteem of Black individuals in the face of negative feedback but conversely harmed the self-esteem of those who received positive feedback. This suggests the efficacy of attributing negative feedback to prejudice as a coping mechanism, while the confounding effect of positive feedback prompts participants to question whether the positivity was provided to avoid any appearance of prejudice from the evaluator. In both cases, ambiguity seems to engender psychological distress.

According to Lazarus and Folkman [9], stress is a transactional process that involves cognitively appraising one’s interpersonal interactions to determine whether well-being is threatened or challenged. Cognitive appraisal determines the degree of the individual stress response. If a situation is deemed threatening or challenging, a secondary appraisal follows to evaluate one’s coping resources and options. However, individuals who are exposed to ambiguous discrimination may struggle to identify threats or challenges, which prevents them from determining the appropriate coping tools to combat the negative interaction, resulting in increased stress. Attributional ambiguity can pose a formidable barrier for individuals seeking to navigate and comprehend negative experiences, particularly due to their uncertainty about whether the cause of these adverse events can be attributed to their own shortcomings or discrimination. This pervasive uncertainty can significantly impede individuals’ access to the necessary resources for coping with the stressors they encounter.

Additionally, ruminating or being bothered about the uncertainty of the situation can further exacerbate the stress experienced by these individuals. Ruminating involves repeatedly thinking about a negative event or experience, which can increase the individual’s focus on the negative aspects of the situation and lead to a negative spiral of emotions [10]. When individuals experience such ambiguity, they may engage in rumination, which involves repetitive and intrusive thoughts about these uncertain situations. This rumination can lead to heightened stress and anxiety as individuals continuously grapple with the unresolved nature of their experiences. Over time, chronic stress and anxiety can contribute to the development or exacerbation of mental health issues, such as depression and anxiety disorders [10–12]. Being bothered (or having negative affect) by the ambiguity of the situation can also lead to heightened feelings of anxiety and distress [13, 14]. When individuals are troubled by this ambiguity, it may be due to a lack of clarity in their social interactions, which, in turn, could lead to heightened emotional responses to the uncertainty. This emotional distress can manifest as increased anxiety, frustration, or even anger, as individuals struggle to make sense of their experiences. Attributional ambiguity can trigger a sense of powerlessness and helplessness, further exacerbating the negative impact on mental health. These mechanisms collectively highlight the importance of addressing attributional ambiguity as a potential stressor and exploring strategies to mitigate its adverse effects on mental well-being in individuals experiencing intergroup interactions. There is limited published research on the impact of ambiguous discrimination on mental health outcomes. The existing experimental studies suggest that uncertainty about whether negative interactions are attributable to racial discrimination or not can negatively affect self-esteem and stress levels, which are known to influence mental health [8, 15, 16]. To date, there are no empirical studies that have quantified the relationship between ambiguous discrimination and depression or overall mental health status.

Individuals from minoritized racial groups may be more likely to experience attributional ambiguity than White populations because they disproportionately endure racial discrimination [17]. However, research on this topic is limited because its complex nature may prevent individuals from identifying and articulating their experiences. Unlike the relatively straightforward nature of quantifying certain forms of overt discrimination in survey research [1] (i.e., experiencing racial profiling by police), attributional ambiguity is more challenging to conceptualize and operationalize, which likely lends itself to being underreported and overlooked. Attributional ambiguity is challenging to capture in survey research compared to overt discrimination because it is often subtle, complex, and context-dependent. Additionally, the language used in survey questions can influence respondents’ interpretations of their experiences, making it difficult to capture experiences of attributional ambiguity.

Our study quantifies attributional ambiguity experiences in a large, diverse sample of U.S. adults. We examine the association between attributional ambiguity and mental health status (depressive symptoms and self-reported mental health status) and hypothesize that attributional ambiguity will be associated with worse mental health. Next, we examine whether the degree of being bothered or the frequency of rumination influences the association between attributional ambiguity and mental health.

## Methods

Data are from the second wave of the “Tufts Equity Survey,” collected between April 23 and May 3, 2021 [18]. The Tufts team contracted a social science research company, IPSOS, to field the survey. IPSOS uses their KnowledgePanel® Service, the oldest and largest online, probability-based panel survey that is designed to be representative of the US adult population. The KnowledgePanel® Service employs rigorous safeguards in an attempt to guard against self-selection bias and facilitate a representative pool of adults. Specifically, the IPSOS sampling frame universe is the United States Postal Service’s Delivery Sequence File (DSF), which captures almost the entire population of the US. From the DSF, a randomly selected sample of US households is mailed an invitation to join the panel. Thus, recruitment is on an invitation-only basis. Of the entire pool of individuals enrolled on the panel, only a certain number are asked to participate in research each month (further promoting reliability and validity by minimizing respondent fatigue.) After initial acceptance of the invitation to join the panel, a comprehensive demographic survey (the Core Profile Survey) is administered; answers to this survey allow efficient panel sampling to reflect those of the US population. The survey was conducted in English and Spanish and IPSOS oversampled Hispanic and non-Hispanic Black individuals by 20%. Complete details regarding survey development and implementation, recruitment, and consent are published elsewhere [18]. We performed a complete case analysis by excluding individuals with missing data for any of the variables of interest. The median time to complete the survey was 15 min. A standard incentive from Ipsos (the cash equivalent of $1) was given to each eligible participant. After completing the survey, participants were entered into a sweepstake to win prizes up to $500. The Institutional Review Board at Tufts University, Medford, USA (protocol 00000428) approved the study protocol.

### Measures

#### Depressive Symptoms

To assess depressive symptoms, we used the 5-item Center for Epidemiologic Studies Depression (CESD) scale. which included questions such as “In the past 7 days, how often have you felt nervous, anxious or on edge?“, “In the past 7 days, how often have you felt depressed?“, and “In the past 7 days, how often have you felt lonely?” Responses were measured on a 4-point scale ranging from 1= “rarely or none of the time” to 4= “all of the time”, with higher scores indicating greater depressive symptoms (scores ranged from 5 to 20).

#### Self-reported Mental Health Status

Mental health status was assessed with a single survey item that asked participants to rate their mental health on a scale ranging from 1=“poor” to 5=“excellent”. Responses were reverse-scored so that a higher score indicates poorer mental health.

#### Attributional Ambiguity

Attributional ambiguity was quantified in three ways: (1) its occurrence, (2) degree of being bothered, and (3) frequency of rumination. First, attributional ambiguity prevalence was assessed by asking, “Sometimes people have things happen to them and they are not sure if the experience was racial discrimination or not. Within the last six months, did you experience a negative situation that you were unsure whether it was racial discrimination or not?” (yes/no). Those who answered “yes” were asked, “How much were you bothered by this event?” to quantify the degree of being bothered with response options ranging from 1=“Not at all” to 5= “To a very great extent”. These respondents were also asked, “How much do you think about this event?” to quantify frequency of rumination. Response options ranged on a 5-point scale from 1="never” to 5="almost always,” with higher values indicating a greater frequency for each construct.

#### Covariates

The following covariates were included in the models given their associations with reporting discrimination: self-reported race/ethnicity (non-Hispanic White, non-Hispanic Black, Hispanic, non-Hispanic Multiracial, non-Hispanic Other), age in years, gender (male or female), education (no high school, high school, some college, bachelor’s degree, or master’s degree), income in USD (less than 10,000; 10,000–24,999; 25,000–49,999; 50,000–74,999; 75,000–99,999; 100,000-149,999; 150,000 and greater, marital status (widowed, divorced, separated, never married), and employment status (full-time, part-time, unemployed).

### Statistical analyses

Descriptive analyses were performed to characterize the sample’s overall sociodemographic characteristics, attributional ambiguity (occurrence, degree of being bothered, and frequency of rumination), and mental health (depressive symptoms and self-reported mental health status). Multiple linear regression models were used to quantify the association between experience of attributional ambiguity and each mental health outcome. The first model tested the association between experiencing attributional ambiguity and depressive symptoms without adjusting for covariates. Next, we adjusted for all covariates. We then restricted the sample to those who reported attributional ambiguity and tested whether being bothered (continuous) and rumination (continuous) were associated with depressive symptoms. The same multivariate models were performed using both mental health outcomes. All analyses were conducted using Stata SE 17.

## Results

**Fig. 1:**
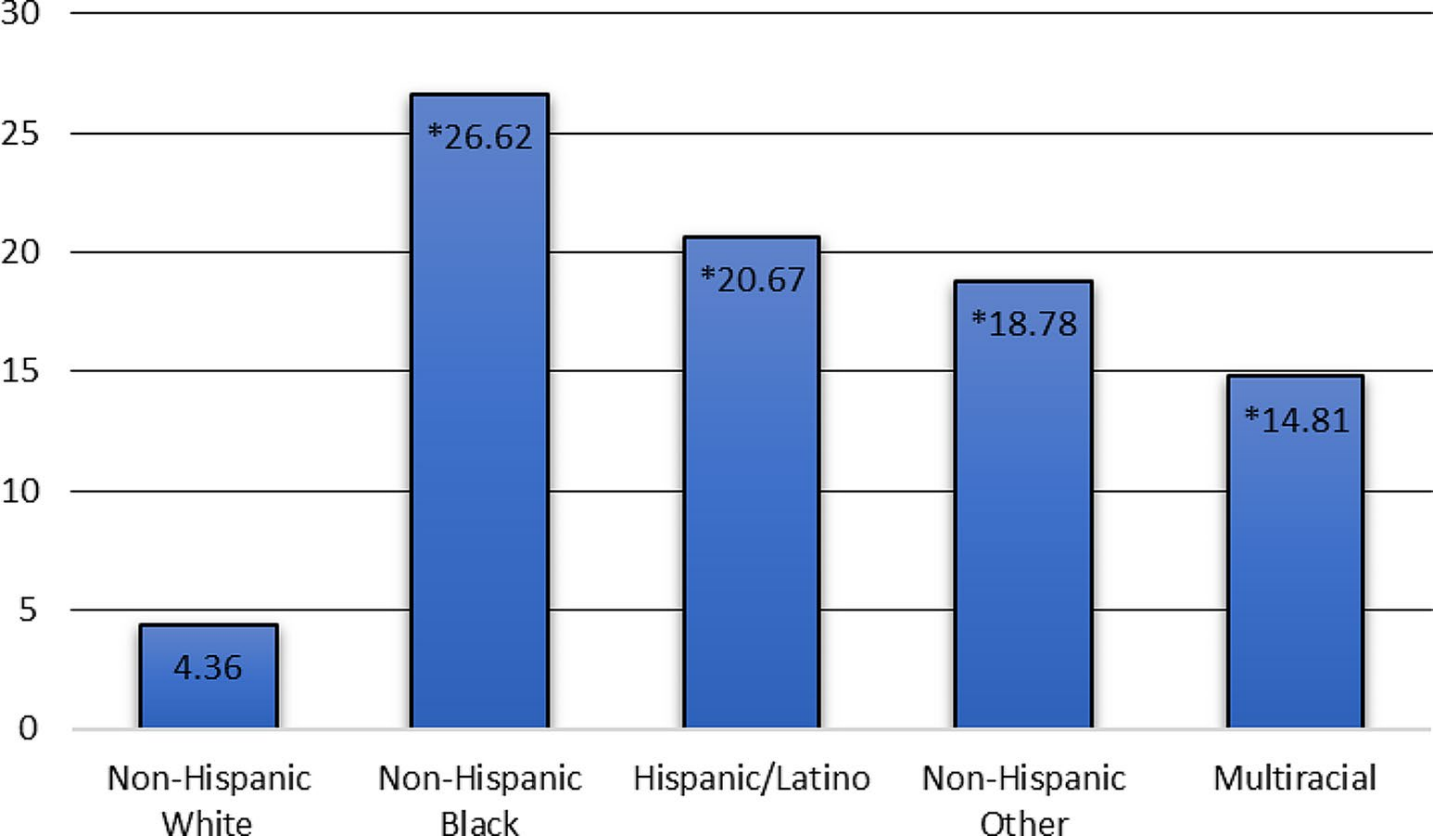
The rate of attributional ambiguity by race/ethnicity * Denotes significant difference at p < 0.05 compared to non-Hispanic White participants

Of the 1,810 respondents with complete data, 22.20% identified as Black, 30.66% as White, 22.37% as Hispanic or Latino of any race, 1.46% as multiracial, and 23.32% as Other Race (Table 1). The sample was 49.55% female with a mean age of 52.10 years and 16.52% of respondents reporting attributional ambiguity experiences. 21% of participants had a total household income above $149,999 and 41.86% held a college or graduate degree. Black (26.62%) and Hispanic (20.67%) participants reported significantly higher rates of attributional ambiguity than White (4.36%) participants (see Fig. 1).

**Table 1:** Descriptive statistics of the Wave 2 Tufts’ Equity in Health, Wealth, and Civic Engagement Survey sample (*N* = 1,810)

	M (SD)
**Age (years)**	52.10 (16.66)
**Depressive symptoms**	9.32 (3.94)
	N (%)
**Self-reported Mental Health Status**	
Poor	11 (3.75)
Fair	46 (15.70)
Good	102 (34.81)
Very good	86 (29.35)
Excellent	48 (16.38)
**Race or Ethnicity**	
White, Non-Hispanic	547 (30.66)
Black, Non-Hispanic	396 (22.20)
Other Race, Non-Hispanic	416 (23.32)
Hispanic	399 (22.37)
Multiracial, Non-Hispanic	26 (1.46)
**Total Household Income**	
Less than $10,000	57 (3.20)
$10,000 to $24,999	141 (7.90)
$25,000 to $49,999	297 (16.65)
$50,000 to $74,999	293 (16.42)
$75,000 to $99,999	274 (15.36)
$100,000 to $149,999	339 (19.00)
$150,000 or more	383 (21.47)
**Rumination**	
Never	34 (11.60)
Seldom	111 (37.88)
Sometimes	95 (32.42)
Often	41 (13.99)
Almost always	12 (4.10)
**Bothering**	
Not at all	13 (4.44)
Very little	51 (17.41)
Somewhat	107 (36.52)
To a great extent	86 (29.35)
To a very great extent	36 (12.29)
**Highest Level of Education**	
No High school diploma or GED	150 (8.39)
High School diploma	438 (24.51)
Some college or associate degree	451 (25.24)
Bachelor’s degree	404 (22.61)
Master’s degree	344 (19.25)
**Marital Status**	
Widowed	78 (4.36)
Divorced	168 (9.40)
Separated	33 (1.85)
Never Married	458 (25.63)
**Employment Status**	
Not working	694 (38.84)
Working part time	224 (12.53)
Working full time	869 (48.63)

### Attributional ambiguity and depressive symptoms

In the unadjusted model, attributional ambiguity was significantly associated with higher depressive symptoms ($\beta$*=1.64; CI: 1.46, 2.13*; Table 2). After adjusting for covariates, attributional ambiguity experience remained significantly associated with depressive symptoms ($\beta$*= 1.47; CI: 0.99, 1.96*; Table 2). Among participants who reported attributional ambiguity, rumination ($\beta$*=0.74; CI: 0.26, 1.22;* Table 3*)* and bother ($\beta$*=0.65; CI: 0.19, 1.11;* Table 4) were significantly associated with greater depressive symptoms.

**Table 2:** Multivariate regression results assessing the association between attributional ambiguity and mental health outcomes (*N* = 1,810)

	Depressive Symptoms $$ \beta $$(95% CI)		Self-reported Mental Health Status $$ \beta $$(95% CI)	
	(1) **Unadjusted**	(2) **Adjusted**	(1) **Unadjusted**	(2) **Adjusted**
**Attributional ambiguity**	1.64 (1.46, 2.13)	1.47 (0.99, 1.96)	0.30 (0.17, 0.42)	0.22 (0.09, 0.35)
**Age (years)**		0.04 (0.03, 0.05)		-0.02 (-0.02, -0.01)
**Race/ ethnicity** ^**a**^				
Non-Hispanic Black Non-Hispanic Other Non-Hispanic, 2 + races		1.18 (0.67, 1.70) 0.73 (0.22, 1.24) -0.48 (-1.96, 0.99)		-0.12 (-0.26, 0.01) 0.11 (-0.02, 0.24) 0.38 (-0.01, 0.76)
Hispanic/Latino		0.08 (-0.44, 0.60)		-0.08 (-0.21, 0.06)
**Female** ^**b**^		-0.73 (-1.09, -0.37)		0.07 (-0.02, 0.16)
**Education** ^**c**^				
High School diploma		0.11 (-0.61, 0.82)		-0.33 (-0.51, -0.14)
Some college or associate degree		-0.13 (-0.86, 0.60)		-0.25 (-0.44, -0.07)
Bachelor’s degree or higher Master’s degree		-0.62 (-1.40, 0.16) -0.86 (-1.68, -0.04)		-0.36 (-0.55, -0.16)
**Income** ^**d**^				
$10,000 to $24,999		0.40 (-0.76, 1.56)		-0.04 (-0.34, 0.26)
$25,000 to $49,999 $50,000 to $74,999 $75,000 to $99,999 $100,000 to $149,999 $150,000 or more		0.70 (-0.39, 1.77) 1.22 (0.12, 2.32) 0.93 (-0.19, 2.05) 1.40 (0.27, 2.52) 1.85 (0.71, 2.99)		-0.12 (-0.40, 0.16) -0.20 (-0.48, 0.09) -0.22(-0.51, 0.07) -0.23 (-0.52, 0.06) -0.40 (-0.70, -0.11)
**Marital Status** ^**e**^				
Widowed Divorced Separated Never Married		-0.49 (-1.39, 0.41) -0.56 (-1.19, 0.08) -1.31 (-2.63, 0.00) -1.05 (-1.55, -0.56)		0.21 (-0.02, 0.44) 0.24 (0.07, 0.40) 0.09 (-0.25, 0.43) 0.17 (0.05, 0.30)
**Employment** ^**f**^				
Working Part-time		-0.42 (-1.00, 0.15)		0.05 (-0.10, 0.20)
Not working		-0.04 (-0.49, 0.40)		0.18 (0.07, 0.30)

^a^ Reference group: White, Non-Hispanic; ^b^ Reference group: Male; ^c^ Reference group: Less than $10,000; ^d^ Reference group: No High school diploma or GED

^e^ Reference group: Widowed; ^f^ Reference group: Working full time

**Table 3:** Multivariate regression results assessing the association between ruminating on attributional ambiguity and mental health outcomes among those who reported attributional ambiguity

	Depressive Symptoms $$ \beta $$(95% CI)		Self-reported Mental Health Status $$ \beta $$(95% CI)	
	(1) **Unadjusted**	(2) **Adjusted**	(1) **Unadjusted**	(2) **Adjusted**
**Rumination**	0.71 (0.23, 1.19)	0.74 (0.26, 1.22)	-0.08 (-0.20–0.04)	-0.05 (-0.18–0.07)
**Age (years)**		0.06 (0.02, 0.10)		-0.01 (-0.02, -0.001)
**Race/ethnicity** ^**a**^				
Non-Hispanic Black Non-Hispanic Other Non-Hispanic, 2 + races		0.97 (-0.93, 2.87) 0.59 (-1.39, 2.57) 1.89 (-2.43, 6.22)		-0.20 (-0.68, 0.29) -0.09 (-0.60, 0.41) 0.21 (-0.90, 1.31)
Hispanic/Latino		-0.28 (-2.24, 1.69)		-0.10 (-0.60, 0.40)
**Female** ^**b**^		-1.17 (-2.16, -0.18)		0.17 (-0.08, 0.42)
**Education** ^**c**^				
High School diploma		1.26 (-0.74, 3.26)		-0.37 (-0.88, 0.14)
Some college or associate degree		2.52 (0.48, 4.55)		-0.45 (-1.00, 0.07)
Bachelor’s degree or higher Master’s degree		1.43 (-0.70, 3.57) 0.88 (-1.33, 3.10)		-0.32 (-0.86, 0.23) -0.03 (-0.60, 0.53)
**Income** ^**d**^				
$10,000 to $24,999		-0.88 (-3.71, 1.96)		0.18 (-0.55, 0.90)
$25,000 to $49,999 $50,000 to $74,999 $75,000 to $99,999 $100,000 to $149,999 $150,000 or more		0.50 (-2.26, 3.26) -0.09 (-2.83, 2.65) -0.75 (-3.59, 2.08) -0.28 (-3.17, 2.62) 0.16 (-2.77, 3.08)		-0.02 (-0.72, 0.69) -0.09 (-0.79, 0.61) -0.17 (-1.00, 0.55) -0.32 (-1.06, 0.42) -0.45 (-1.19, 0.30)
**Marital Status** ^**e**^				
Widowed Divorced Separated Never Married		-0.63 (-3.15, 1.89) -0.36 (-2.10, 1.38) -3.00 (-6.31, 0.32) -0.30 (-1.59, 0.98)		-0.24 (-1.00, 0.40) 0.18 (-0.26, 0.63) -0.05 (-0.89, 0.80) 0.38 (0.06, 0.71)
**Employment** ^**f**^				
Working Part-time		-0.04 (-1.54, 1.46)		-0.02 (-0.40, 0.37)
Not working		0.54 (-0.69, 1.78)		0.15 (-0.16, 0.46)

^a^ Reference group: White, Non-Hispanic; ^b^ Reference group: Male; ^c^ Reference group: Less than $10,000; ^d^ Reference group: No High school diploma or GED

^e^ Reference group: Widowed; ^f^ Reference group: Working full time

**Table 4:** Multivariate regression results assessing the association between being bothered by attributional ambiguity and mental health outcomes among those who reported attributional ambiguity

	Depressive Symptoms $$ \beta $$(95% CI)			Self-reported Mental Health Status $$ \beta $$(95% CI)	
	(1) **Unadjusted**	(2) **Adjusted**		(1) **Unadjusted**	(2) **Adjusted**
**Degree of being bothered**	0.63 (0.16, 1.09)	0.65 (0.19, 1.11)		-0.07 (-0.19, 0.05)	-0.05 (-0.17, 0.07)
**Age (years)**		0.06 (0.02, 0.10)			-0.01 (-0.02, -0.00)
**Race/ethnicity** ^**a**^					
Non-Hispanic Black Non-Hispanic Other Non-Hispanic, 2 + races		0.90 (-0.10, 2.81) 0.32 (-1.66, 2.31) 1.56 (-2.76, 5.89)			-0.19 (-0.68, 0.29) -0.07 (-0.58, 0.43) 0.23 (-0.87, 1.33)
Hispanic/Latino		-0.42 (-2.39, 1.55)			-0.09 (-0.59, 0.41)
**Female** ^**b**^		-1.10 (-2.10, -0.10)			0.16 (-0.10, 0.42)
**Education** ^**c**^					
High School diploma		1.09 (-0.91, 3.09)			-0.36 (-0.86, 0.15)
Some college or associate degree		2.34 (0.31, 4.37)			-0.44 (-0.96, 0.08)
Bachelor’s degree or higher Master’s degree		1.41 (-0.73, 3.56) 0.77 (-1.45, 2.98)			-0.32 (-0.86, 0.23) -0.03 (-0.59, 0.54)
**Income** ^**d**^					
$10,000 to $24,999		-0.56 (-3.39, 2.27)			0.16 (-0.56, 0.88)
$25,000 to $49,999 $50,000 to $74,999 $75,000 to $99,999 $100,000 to $149,999 $150,000 or more		0.86 (-1.90, 3.62) 0.29 (-2.43, 3.01) -0.48 (-3.31, 2.35) 0.00 (-2.88, 2.89) 0.46 (-2.45, 3.38)			-0.04 (-0.74, 0.66) -0.12 (-0.81, 0.57) -0.19 (-0.91, 0.53) -0.34 (-1.07, 0.39) -0.47 (-1.21, 0.27)
**Marital Status** ^**e**^					
Widowed Divorced Separated Never Married		-0.78 (-3.31, 1.76) -0.55 (-2.29, 1.19) -3.22 (-6.55, 0.11) -0.42 (-1.71, 0.87)			-0.23 (-0.88, 0.41) 0.20 (-0.25, 0.64) -0.03 (-0.88, 0.81) 0.39 (0.07, 0.72)
**Employment** ^**f**^					
Working Part-time		-0.16 (-1.67, 1.35)			-0.01 (-0.39, 0.38)
Not working		0.46 (-0.78, 1.69)			0.16 (2.08, 4.37)

^a^ Reference group: White, Non-Hispanic; ^b^ Reference group: Male; ^c^ Reference group: Less than $10,000; ^d^ Reference group: No High school diploma or GED

^e^ Reference group: Widowed; ^f^ Reference group: Working full time

### Attributional ambiguity and self-reported mental health status

Attributional ambiguity was significantly associated with poorer self-reported mental health status in both unadjusted ($\beta$*=0.30; CI: 0.17–0.42;* Table 2) and adjusted models ($\beta$*=0.22; CI: 0.09–0.35*). We did not find evidence that rumination and bothered were significantly associated with self-reported mental health status.

We explored whether the association between attributional ambiguity and mental health outcomes varied by race/ethnicity. Our analysis did not find any evidence that the associations significantly varied by race/ethnicity.

## Discussion

Attributional ambiguity– the difficulty in determining whether a negative experience is due to discrimination– may be an important mechanism behind racial disparities in mental health. As a psychosocial stressor, it is an important component of the discrimination experiences of racially minoritized individuals [15]. We found that attributional ambiguity was common, and Black and Hispanic participants experienced it more than White participants. Experiencing attributional ambiguity was associated with higher levels of depressive symptoms and poor mental health status. The magnitude of bother and rumination were significantly and positively associated with depressive symptoms. Taken together, our findings suggest that the emotional impact of attributional ambiguity may contribute to poor mental health outcomes and that minoritized populations may be particularly threatened.

Our study was the first to quantify the phenomenon of attributional ambiguity and measure its frequency and influence on two indicators of mental health (depressive symptoms and self-reported mental health) in a diverse sample of US adults. Our findings are consistent with previous experimental studies that have shown that the experience of ambiguous discrimination can negatively impact psychological processes such as self-esteem and increase negative affect. For instance, Crocker and colleagues found that Black participants exhibited lower self-esteem when they received negative feedback from White evaluators whom they believed knew their race compared to those who believed their White evaluators did not know their race [8]. The condition where White evaluators knew the participant’s race may have induced attributional ambiguity, whereby the participant did not know whether the negative feedback was due to their performance or race. Another study revealed that, even in the presence of positive feedback, Black participants demonstrated a sense of caution and exhibited defensive responses, underscoring the intricate nature of acceptance within interracial interactions and its potential ramifications for health outcomes. It also implies that attributional ambiguity may not solely result from negative experiences; conversely, positive experiences can engender a similar sense of ambiguity, especially in interactions involving individuals from out-groups. Therefore, it is important for future studies to explore other ways to capture attributional ambiguity in surveys, with a focus on the role of the perpetrator.

Rumination and feeling bothered by the uncertainty can act as ways in which attributional ambiguity impacts our mental well-being. When people are dealing with ambiguity, they might find themselves repeatedly mulling over the negative event. This habit of overthinking can intensify negative emotions, possibly kickstarting a harmful emotional cycle [10]. Experiencing a negative emotional response (or feeling bothered) due to the uncertainty surrounding the event can likewise intensify feelings of distress [13, 14]. The biobehavioral mechanisms linking these responses to mental health remain unclear, we provided a solid foundation for future avenues of investigation. For example, researchers could identify behavioural (i.e., sleep hygiene), biological (i.e., the stress hormone cortisol), and environmental (i.e., living in a racially segregated neighbourhood) pathways linking ruminating and being bothered to mental health. Moreover, we used a validated scale for depressive symptoms and a self-reported mental health item to quantify mental health but future studies could use expanded measures, such as anxiety and externalizing disorders, to gain a more comprehensive understanding of the relationship between attributional ambiguity and mental health.

We found that approximately 16% of the overall sample experienced attributional ambiguity in the past six months, and Black (26.62%) and Hispanic (20.67%) participants reported higher rates of attributional ambiguity than White (4.36%) participants. Future qualitative research could be conducted to capture the complexity and diversity of attributional ambiguity experiences and inform additional quantitative survey items. The means by which people comprehend ambiguous discrimination, the cognitive coping processes, and the long-term implications of ambiguous situations are complex yet unclear. Such knowledge could garner actionable insight to measure and address this important psychosocial stressor.

### Limitations

This study was cross-sectional; thus, causality could not be established. Future research should apply a longitudinal design to firmly establish the relationship between attributional ambiguity and mental health. Our sample was conducted among highly educated individuals with high incomes. Future research with sufficient sample sizes to ensure statistical power should examine the relationship between attributional ambiguity across diverse sociodemographic categories to identify (1) whether attributional ambiguity occurs in lower income populations, where discrimination experiences may be more salient, and (2) to identify subgroups at greater risk of attributional ambiguity. Discrimination can contribute to increased experiences of ambiguity. To estimate the independent association between ambiguity and mental health more accurately, adjusting for discrimination may be necessary. However, we did not adjust for discrimination in this study because the current measure of discrimination in the Tufts University Equity in Health, Wealth, and Civic Engagement Study asks about perceived unfair treatment within the past 12 months. Adjusting for discrimination exposure this way would introduce overadjustment bias and collider bias, given how discrimination and ambiguity exposure are measured. People exposed to ambiguity in the past six months may perceive discrimination in the last month, leading to poorer mental health. It is also plausible that mental health can contribute to perceived discrimination, which would contribute to collider bias. Research using longitudinal data would be able to disentangle the causal pathway among ambiguity, discrimination, and mental health and gain a better understanding of the independent association between ambiguity and mental health. The current survey categorized some groups as 2 + race and non-Hispanic Other, which made up a considerable proportion of the study sample, limiting our ability to identify other racial and ethnic groups. By expanding the categories, future studies can better capture the nuanced experiences of individuals from diverse backgrounds. Last, this study only focused on one aspect of attributional ambiguity related to racial discrimination. Other marginalized groups, such as women, sexual minorities, immigrants, and those from lower social classes may experience unique forms of attributional ambiguity with differing effects on mental health. Simultaneously, special attention should be given to those with multiple marginalized identities.

## Conclusion

Our study adds to growing body of literature that Black and Hispanic individuals experience higher levels of attributional ambiguity than their White counterparts. Our findings indicated that experiencing attributional ambiguity within the past six months was associated with greater depressive symptoms and poorer self-reported mental health status. Rumination and bother from attributional ambiguity increased the magnitude of depressive symptoms. Ambiguous discrimination is a critical aspect of the daily experience of racially minoritized group members that need greater attention in health research.

## Data Availability

De-identified data from Tufts University Equity in Health, Wealth, and Civic Engagement Study can be obtained by contacting Dr. Jennifer Allen (Jennifer.allen@tufts.edu).
